# Effects of Maturation on Antibacterial Properties of Vietnamese Mango (*Mangifera indica*) Leaves

**DOI:** 10.3390/molecules29071443

**Published:** 2024-03-23

**Authors:** Hai Thanh Nguyen, Atsushi Miyamoto, Hao Thanh Hoang, Tra Thi Thu Vu, Pitchaya Pothinuch, Ha Thi Thanh Nguyen

**Affiliations:** 1 Department of Plant Biotechnology, Faculty of Biotechnology, Vietnam National University of Agriculture, Trau Quy Crossing, Gia Lam District, Hanoi 100000, Vietnam; nthaicnsh@vnua.edu.vn; 2 Department of Veterinary Pharmacology, Joint Faculty of Veterinary Medicine, Kagoshima University, 1-21-24 Korimoto, Kagoshima 890-0065, Japan; k1330977@kadai.jp; 3 Department of Veterinary Pharmacology, Faculty of Veterinary Medicine, Vietnam National University of Agriculture, Trau Quy Crossing, Gia Lam District, Hanoi 100000, Vietnam; 650537@sv.vnua.edu.vn (H.T.H.); vutra@vnua.edu.vn (T.T.T.V.); 4 Faculty of Food Technology, Rangsit University, 52/347 Muang-Ake Phahonyothin Road, Lak-Hok, Pathumthani 12000, Thailand; pitchaya.p@rsu.ac.th

**Keywords:** *Mangifera indica*, leaf, maturity, mangiferin, antibacterial effect

## Abstract

This study, for the first time, has investigated the relationships between alterations of mangiferin contents in mango leaves at different maturity stages and their antibacterial properties. Leaves were classified into six different maturity stages based on their color: (1) young dark reddish brown, (2) young yellow, (3) young light green, (4) mature green, (5) old dark green, and (6) old yellow leaves. Ethanol extracts were then examined against Gram-positive and Gram-negative bacteria, applying broth dilution and agar well diffusion methods. In addition, we also measured the mangiferin contents in leaves at different stages for the purpose of evaluating how the changes in this phytochemistry value affects their activities against bacteria. The results showed that extracts from leaves at young ages had better antibacterial properties than those from old leaves, as evidenced by the lower minimum inhibitory concentrations and larger inhibitory zones. In addition, we also found that the contents of mangiferin were significantly decreased followed the maturation process. These results suggest that mango leaves at young stages, especially dark reddish brown and young yellow leaves, are preferable for application in bacterial infections and other therapies related to mangiferin’s constituents.

## 1. Introduction

In traditional medicine, when leaves of a plant are applied as herbal medicine, it is generally advised to collect the materials when they are fully developed and at peak maturity, described as “not young, not old”, because this level of maturation is believed to have high therapeutic properties [1]. However, in mangoes, leaves at young ages are preferable for the treatment of diabetes, hypertension, anxiety, bile or kidney stones, respiratory disease, and bacterial infections [2,3,4]. When applied as tea or vegetables in Vietnam, leaves of young ages are also selected [4]. Similarly, as part of a folk tradition in India and Thailand, when a high-fat diet is consumed, young mango leaves are often consumed alongside it for health [5]. Many researchers have chosen young leaves of mangoes in the investigation of their therapeutic properties, but they have not described in detail the traditional or scientific bases of this choice [6,7,8,9,10,11]. Several studies have examined the relationship between mango leaves’ stages of maturity and their pharmacological functions, such as inhibition of pancreatic lipase [12], inhibition of advanced glycation end-products [13], antioxidant activities [14,15], and allelopathic [16] and antibacterial effects [17,18]. Changes in phytochemical profiles due to maturation, including the alterations of bioactive compounds, have been reported to mediate the differences in several therapeutic properties of mango leaves, including the inhibition of pancreatic lipase [12], antioxidant activities [15], and allelopathic functions [16]. However, regarding their efficacy against bacteria, there have been no studies investigating how alterations in the phytochemical constituents of this material at various maturity stages contribute to its different activities. Therefore, the mechanisms responsible for its modes of action remain undetermined [17,18]. 

While changes in color during the maturation process of mangoes can mainly be explained by alterations in their pigment composition, researchers have also found that there are significant differences in many other phytochemical constituents, including both nutritional ingredients and bioactive compounds, which result in mangoes’ usage as both food or medicine [13,19]. Among the various bioactive compounds in mangoes, mangiferin has been identified as the major and the most abundant ingredient [20]. This secondary metabolite is not only important in the defense mechanisms of plants against pathogenic microorganisms but also plays vital roles in a wide range of pharmacological activities [21,22]. In addition, it has been identified as the principal compound responsible for the antibacterial properties of mango leaves [23,24,25,26,27]. While previous studies have observed that mango leaves at different maturity stages have different effects on bacteria [17,18], they have not yet determined the changes that occur in the phytochemical constituents of tested materials. Therefore, we also measured the mangiferin contents of leaves in order to verify if changes in the concentration of this compound are responsible for leaves’ different antibacterial properties.

## 2. Results and Discussion

### 2.1. Effects of Maturity Stages on Mangiferin Contents of Mango Leaves

**Figure 1:**
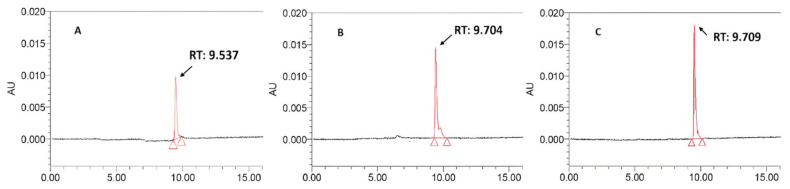
Representative HPLC chromatography of mangiferin standards at different concentrations. (**A**): mangiferin 0.4 mg/mL, (**B**): mangiferin 0.6 mg/mL, and (**C**): mangiferin 0.8 mg/mL.

Representative HPLC chromatograms of mangiferin standards are shown in Figure 1.

The retention time (RT) of mangiferin was from 9.537 min (Figure 1A) to 9.709 min (Figure 1C). Linearity was established by comparing the peak areas with mangiferin standard concentrations. The calibration curve can be represented by the equation y = 106,818x + 4984.8 (R^2^ = 0.9925) for mangiferin (where y is the peak response area and x is the concentration). 

**Figure 2:**
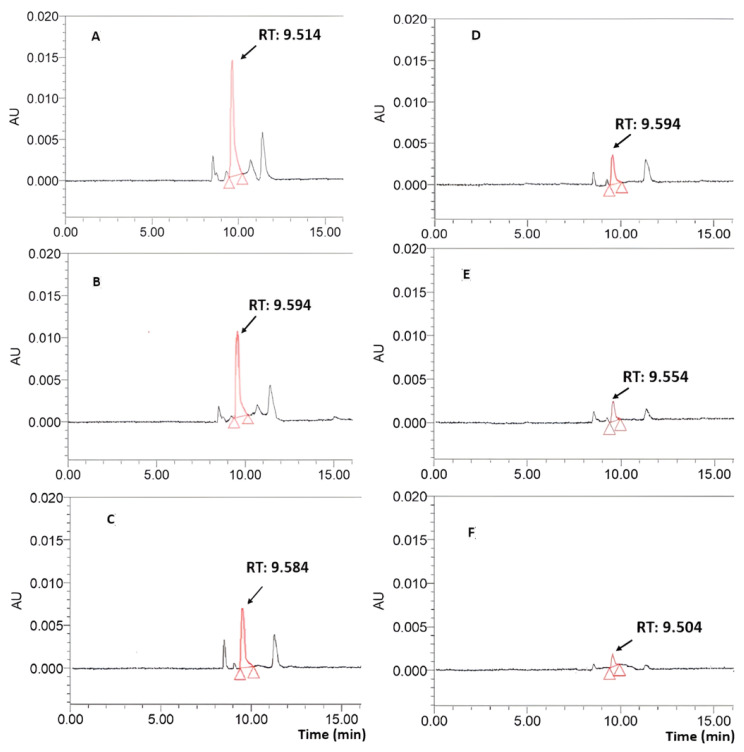
Representative HPLC chromatography of mango leaf ethanol extracts (2 mg/mL) at different maturity stages. (**A**): Young dark reddish brown leaf, (**B**): young yellow leaf, (**C**): young light green leaf, (**D**): mature green leaf, (**E**): old dark green leaf, and (**F**): old yellow leaf. The x axis represents retention time, and the absorbance unit on the y axis represents absorbance units (a signal corresponding to the response created by the detector) at 360 nm.

Representative HPLC chromatograms of leaf extracts at six different maturity stages are shown in Figure 2.

The peaks in RT that varied from 9.504 to 9.594 min in the chromatography of leaf extracts were identified as belonging to mangiferin. We compared the area of these peaks with that of the standard; we also compared the mangiferin contents of mango leaves at different maturation stages, which were calculated based on the weights of the extracts (mg/g extract) or initial leaf materials (mg/g dried powder). These are shown in Table 1.

From Table 1, we observed that the yields of mango leaves varied between 26.39 and 43.66%. These results were in accordance with the previous research of Chewchinda et al. [28], who determined yields of crude ethanol extracts from mango leaves within a range from 25.26 to 47.44% depending on plant variety. When converted to dried weights of initial materials, contents of mangiferin were identified within a range from 8.71 to 81.82 mg/g dried powder, which is equal to 0.87 to 8.18%. These results were similar to but slightly lower than those reported by Stohs et al. [29], who reported that mangiferin contents in mango leaves varied from 2 to 15% depending on varieties and geographic sources. In our study, the mangiferin content of young dark reddish brown leaves was 81.82 mg/g, in accordance with the report of Parafati et al. [30], who also investigated red-colored mango leaves and reported the concentration to be 84.52 mg/g dried weight. In young yellow leaves, the mangiferin content was determined to be 73.12 mg/g, which is higher than that reported by Barreto et al. [31], who determined the content of generally young leaves to be 58.12 mg/g. Similarly, when converted to the weight of crude extracts, mangiferin contents in young dark reddish brown and young yellow leaves were 310.06 and 220.71 mg/g extract, respectively; these values are higher than the results of Chewchinda et al. [28], who determined that these contents ranged from 105.18 to 197.32 mg/g extract. In young light green leaves, mangiferin content was determined to be 60.23 mg/g dried powder and 137.96 mg/g extract, similar to the results of Barreto et al. [31] and Chewchinda et al. [28], who, respectively, reported this content to be 58.12 mg/g dried powder and 105.18 to 197.32 mg/g extract. The higher contents observed in our studies for dark reddish brown and young yellow leaves might be partly explained by the fact that Chewchinda et al. [28] and Barreto et al. [31] classified all newly generated leaves as one group of young leaves, while our study further divided them into three smaller groups to examine separately. Mangiferin contents in old dark green leaves were determined at 13.36 mg/g dried weight, being lower than the study of Das et al. [32], who reported this concentration to be 36.9 mg/g. In contrast, this was higher the result of Loan et al. [33], who identified the content to be 9.51 mg/g. The differences might be explained via the fact that mangiferin constituents in mango leaves are largely dependent on origin and plant variety [28,29]. 

From Table 1, it is evident that there was a significant decreasing trend in mangiferin concentrations following the leaf maturation process. These results are in accordance with previous studies [34,35]. In addition, the decrement was more obvious in late maturity (from green to old yellow leaves). For example, the mangiferin content in mature leaves decreased to less than 50.0% of the amounts detected in leaves at the first three stages. Specifically, it was equal to only 32.0% (26.20 mg/g vs. 81.82 mg/g), 35.8% (26.20 mg/g vs. 73.12 mg/g), and 43.5% (26.20 mg/g vs. 60.23 mg/g) of that in young dark reddish brown, young yellow, and young light green leaves, respectively. This reduction continued when the materials reached the older stages, as the content in the old yellow color group was observed to equal only 33.2% (8.71 mg/g vs. 26.20 mg/g) and 10.6% (8.71 mg/g vs. 81.82 mg/g) of the amounts detected in mature and young dark reddish brown leaf groups. These results were in accordance with a previous study [34], which also reported that mangiferin contents reduced by more than 50% when the leaves reached their withering yellow-color stage. A similar phenomenon was also observed in other parts of mango plants, such as in pulp and peel, because their amounts of mangiferin were remarkably decreased when the fruits were over-ripened [36]. Studies on many plants have found that most secondary metabolites showed decreasing patterns in terms of development stages and senescence [37]. This shift could be explained via the mobilization of recycling of nutrients from old leaves to sinks, such as senescing leaves or developing seeds [38], which leads to decrements in the accumulation of various secondary metabolites during plants’ later maturation [37,39,40]. 

### 2.2. Effects of Maturity Stages on Antibacterial Effects of Mango Leaves

#### 2.2.1. Effects of Maturation on Minimum Inhibitory Concentration (MIC) Values of Mango Leaf Ethanol Extracts

The MIC values of ethanol extracts of mango leaves at six maturity stages and mangiferin against six bacteria are shown in Table 2.

From Table 2, we observed that mango leaves exerted effects on both Gram-positive and Gram-negative bacteria. Similarly to our results, several studies have also observed that extracts from mango materials show effects on both Gram-positive bacteria, such as *B. cereus*, *Bacillus megaterium*, *B. subtilis*, *S. aureus*, *Staphylococcus epidermidis*, *Streptococcus agalactiae*, *Streptococcus faecalis*, *Streptococcus pneumonia*, and *Streptococcus pyogenes* [3,41,42,43,44,45,46,47], and Gram-negative ones, including *E. coli*, *Klebsiella pneumonia*, *Proteus mirabilis*, *Proteus vulgaris*, *P. aeruginosa*, *Pseudomonas fluorescens*, *Salmonella typhi*, *Shigella flexneri*, and *Shigella sonnei* [3,41,42,43,44,45,46,47]. The MIC values of mango leaves varied between 1.95 and 1000 μg/mL, similar to some previous studies [46,48,49], while they were occasionally higher than those reported by others [41,50]. The most obvious reason for the differences in results may be that mango leaves’ effects depend up on many factors like origin, type of processing, and the bacterial strains investigated [51]. According to the classification established by Kuete and Efferth [52], the antibacterial activity of a plant extract is considered strong when MIC values are below 100 μg/mL, moderate when 100 ≤ MICs ≤ 625 μg/mL, and weak when MICs > 625 μg/mL. Consequently, the ethanol extracts from mango leaves of all three young- and mature-stage leaves examined in this study could be considered to have moderate-to-strong activities against all bacteria (with MICs ranging from 1.95 to 500 μg/mL), while old leaves, both of a dark green and old yellow color, showed only weak effects against *P. aeruginosa* ATCC 9027 (MICs were 1000 μg/mL). 

Similarly to leaf extracts, our study reveals that mangiferin has a wide range of antibacterial properties as it was active against both Gram-positive and Gram-negative bacteria. These observations were in accordance with several previous reports [20,21,22,53]. The MIC values of mangiferin were found to vary from 3.91 to 62.5 μg/mL, similar to the results of Singh et al. [21] and Yehia and Altwain [27] and lower than those reported by Savikin et al. [54], who determined that these MIC values ranged from 202 to 310 μg/mL. This could be partly explained by the differences in the examined microorganisms, because isolated mangiferin has been observed to have very selective effects on bacterial species [21,49]. 

When comparing MICs obtained from extracts from six leaf groups, it is noticeable that there is trend of increasing MIC values following growth to mature stages, as those values from younger samples were always lower than those of old samples, regardless of examined bacterial strains. This decrement might be partly explained by a reduction in mangiferin contents, as this compound was also observed to exert inhibitory effects against all bacteria in the same set of experiments (MICs ranged from 1.95 to 62.5 μg/mL) (Table 2). In addition, the sensitivity and resistance of each bacterial strain to extracts and mangiferin were correlated. For examples, *B. cereus*, *S. aureus*, and *E. coli* were more sensitive to both leaf extracts and mangiferin, as evidenced by their lower MIC values. In contrast, *B. subtilis*, *S.* typhimurim, and *P. aeruginosa* were more resistant, as higher concentrations of the tested materials were required to reach their MICs (Table 2). 

Among all investigated bacteria, *S. aureus* ATCC25923 was the most sensitive to both mango leaves and mangiferin, as evidenced by its lowest MIC values from extracts of all six leaf maturity stages and the isolated compound; among them, the MICs of the former were ranged from 1.95 to 125 μg/mL, while that of the later was 3.91 μg/mL. The results were similar to those of several previous studies, which also applied broth dilution methods to investigate and reported the high sensitivity of *S. aureus* to mango materials and mangiferin, as MICs against this bacterial species were lowers than those against other bacteria [27,41,45,46]. In contrast, *P. aeruginosa* ATCC 9027 was the most resistant, because the MICs of all leaf extracts and mangiferin against this bacterium were always higher than those against other species (Table 2). This observation is in accordance with the reports of Dzotam et al. [49], because *P. aeruginosa* was also observed to be highly resistant to extracts from mango leaves, as evidenced by its MIC values (1024 μg/mL), which were the highest among all investigated bacteria. 

Interestingly, we observed that MICs of extracts from leaves at the two youngest stages (dark reddish brown and yellow color) were able to exert better activities than purified mangiferin against *S. aureus*, as evidenced by the lower MICs (1.95 vs. 3.91 μg/mL). No previous studies have performed a comparative investigation of the MIC values of mango leaf extracts and purified mangiferin, but the superior therapeutic effects of whole extracts on this isolated compound have been observed with other pharmacological functions [23,55]. For examples, Alañón et al. [55] and Sanchez et al. [56] reported that although mangiferin appeared to play a key role in the therapeutic functions of mango materials, whole crude extracts could exert superior effects (such as in antiplatelet aggregation effects [55], in vitro scavenging activities, and also in vivo inhibition of tissue damage induced by oxidative stress [56]), compared to those produced by isolated mangiferin when applied at the same concentrations, suggesting the important roles of other constituents in the total properties of this plant. From our results, it is also possible to suggest that compounds different from mangiferin are significantly involved in antibacterial effects of mango leaves, at least in the case of *S. aureus*.

#### 2.2.2. Effects of Maturation on Inhibitory Zones of Mango Leaf Ethanol Extracts

**Figure 3:**
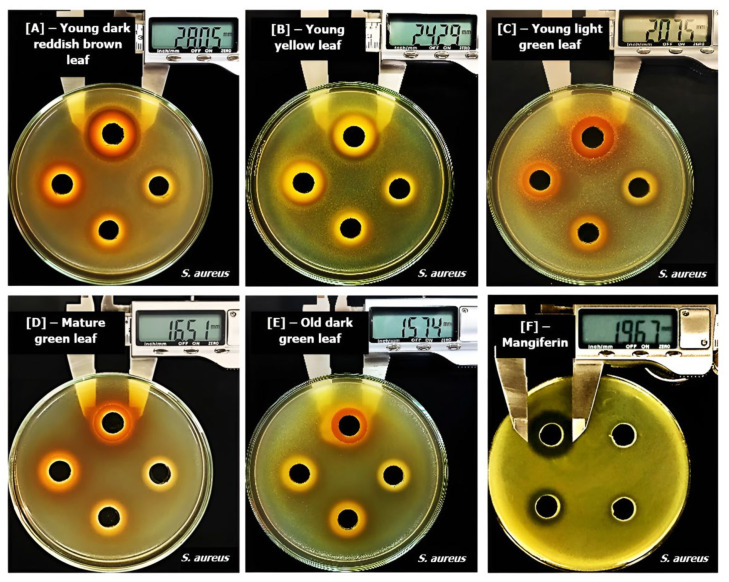
Inhibitory zone (mm) induced by ethanol extracts from leaves at different maturity stages and mangiferin on *Staphylococcus aureus* ATCC25923. (**A**): Young dark reddish brown leaf, (**B**): young yellow leaf, (**C**): young light green leaf, (**D**): mature green leaf, (**E**): old dark green leaf and (**F**): mangiferin.

**Figure 4:**
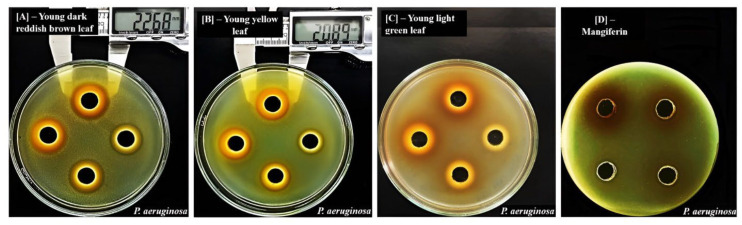
Inhibitory zone (mm) induced by ethanol extracts from leaves at different maturity stages and mangiferin on *Pseudomonas aeruginosa* ATCC9027. (**A**): Young dark reddish brown leaf, (**B**): young yellow leaf, (**C**): young light green leaf and (**D**): mangiferin.

Inhibitory zones (mm) induced by ethanol extracts of mango leaves at different maturity stages and mangiferin on bacteria are shown in Table 3 and Figure 3 and Figure 4.

From Table 3, we observed that mango leaf extracts were able to induced effects on both Gram-positive and Gram-negative bacteria, confirming the results obtained with the broth dilution method. When comparing the effects of leaves at different stages, we observed that maturation processes clearly reduced their antibacterial properties, as shown by the significantly smaller inhibitory zones against both Gram-positive and Gram-negative bacteria. Young dark reddish brown and young yellow-colored leaves produced the best effects, as they exerted inhibition on all six investigated bacteria; they were more effective than young light green leaves, as these materials showed effects on only five bacteria. Mature and old dark green leaves were less potent, as they were able to induce inhibitory zones only against *S. aureus* (Figure 3). Old yellow leaves had the weakest activities, as they showed no inhibition against all six bacteria. These results suggest that leaves at early maturity stages, especially those of a dark reddish brown and yellow color, are better materials in terms of antibacterial effects. Similarly to the results from the broth dilution methods, a trend of reduction in activities following maturation processes was also observed, because the diameters of inhibitory zones were found to be significantly decreased against all bacteria. This reduction could be attributed, at least in part, to the decrement of mangiferin contents, as this compound was also able to produce significant inhibitory zones against bacteria in the same set of experiments. Specifically, test results with mangiferin showed that at concentrations from 0.25 to 0.5 mg/mL, the compound was able to induce effects against five bacteria, including *B. cereus*, *B. subtilis*, *S. aureus*, *E. coli*, and *S. typhimurium* (Table 3). When normalizing the concentrations of extracts applied in each well of this well diffusion method to the contents of mangiferin, the range of examined doses from 2 to 0.25 mg/mL was converted into 0.62–0.08 mg/mL for young dark reddish brown, 0.44–0.06 mg/mL for young yellow, 0.28–0.03 mg/mL for light green, 0.13–0.02 mg/mL for mature green, 0.06–0.01 mg/mL for old dark green, and 0.04–0.01 mg/mL for old yellow leaves. By comparing these normalized concentrations with those of mangiferin that induced significant inhibitory zones, we concluded that the mangiferin contents are responsible, at least in part, for the effects of the young dark reddish brown, young yellow, and young light green leaves against *B. cereus*, *B. subtilis*, *S. aureus*, *E. coli*, and *S. typhimurium*. In contrast, the effects of leaves on *P. aeruginosa* ATCC 9027 require the important roles of other compounds, as mangiferin alone did not show any inhibition against this bacterium, but whole extracts from young dark reddish brown and young yellow leaves were able to have significant effect (Figure 4). However, it is still not possible to rule out the roles of this compound in the effects of leaf extracts against *P. aeruginosa*, as mangiferin has been reported not only to directly inhibit bacteria but also to act synergistically with other co-existing compounds and enhance their effects [57,58,59]. 

Similarly to the results of broth dilution methods, mango leaves and mangiferin compound had the greatest effect on *S. aureus* ATCC 25922, as evidenced by the significantly larger inhibitory zones than those obtained with other bacteria. In addition, only *S. aureus* was sensitive to the leaves of five maturity stages, including the mature green and old dark green materials (Figure 3); meanwhile, other bacteria were only sensitive to leaves at the three young stages (Table 3). These results are similar to those of several previous studies, which also applied agar well diffusion methods to investigate and reported the high sensitivity of *S. aureus* to mango materials [41,45,60]. Researchers also proposed this herbal plant as a source of novel antibacterial compounds to combat pathogenically isolated methicillin-resistant *S. aureus*, which have which has become a global problem due to nosocomial infections [61]. Extracts from mangoes have been observed not only to inhibit the growth of *S. aureus* [62] but also to reduce their biofilms [50] and synergize with many antibiotics to produce better effects on this bacterial species [63]. Our study, together with previous reports, highlights the potential of applying mango leaves as a natural therapy to control *S. aureus* species, the development of which has become a massive concern in clinical practice due to their multi-drug resistance [64].

In contrast to *S. aureus*, *P. aeruginosa* ATCC 9027 was found to be the most resistant to both mango leaves and mangiferin, as this bacterium was inhibited only by the extracts from leaves from the two youngest stages (dark reddish brown and yellow color) and was resistant to both young light green leaves and mangiferin (Figure 3). The other five examined bacteria were all sensitive to the two later materials (Table 3). This result was similar to those observed in broth dilution methods, confirming the strain’s resistance. In addition, it was in accordance with a previous study [21], which also applied diffusion methods to investigate and reported the high resistance of *P. aeruginosa* against mangiferin, because while the compound could induce significant inhibitory zones on all other tested bacteria, including *Bacillus pumilus*, *B. cereus* and *Salmonella virchow*, it was not able to produce any effects on *P. aeruginosa*. 

When comparing the effects of whole extracts with mangiferin, it was noticeable that regardless of examined bacteria, young dark reddish brown and young yellow leaves were always able to produce inhibitory zones that were significantly larger than those obtained with isolated compounds (Table 3 and Figure 3 and Figure 4). These results were similar to the observations in broth dilution methods for *S. aureus*. The lesser effectiveness of pure isolated compounds compared with crude mixtures from whole materials suggests that the effects cannot be solely attributed to a bioactive compound, and synergism among various ingredients is required for maximum effects [23]. Several previous studies have established mangiferin as the possible active principle of mango materials and have attributed most of the biological effects of the extracts, including their antibacterial functions, to this component [23]. However, researchers also suggest that the total antibacterial properties of mango leaves might involve the important roles of others compounds, such as tannins, which were also highly potent against bacteria [50]. In addition, the concept that whole plant materials are more advantageous than isolated active ingredients also underpins the philosophy of traditional herbal medicine [65]. 

Though researchers have previously observed that the plant parts of mango trees that had higher contents of mangiferin could produce better effects against bacteria [27,59], our study is the first one to investigate the relationships between changes in the mangiferin contents of leaves due to maturation and their antibacterial properties. Taken altogether, our study reveals that differences in maturity stages significantly alter the antibacterial effects of mango leaves, which could be explained, at least in part, via the decrement in mangiferin contents. In addition, the results also highlight young mango, characterized by a color ranging from dark reddish brown to light green, as the best materials to fight bacteria. Investigation into leaves of other herbal plants has also revealed similar results, showing that in the comparison with mature or old leaves, young leaves generally exhibit higher contents of bioactive compounds and produce stronger antibacterial effects [66]. However, in the case of mango leaves, relationships between maturity levels and therapeutic properties seem to be varied due to examined biological functions. For example, while younger leaves are preferred for the inhibition of pancreatic lipase [12], old leaves have been observed to exert better properties in the inhibition of advanced glycation end-products [13], antioxidant activities [14], and allelopathic effects [16]. Therefore, we suggest that the influences of maturation on the pharmacological properties of mango leaves need to be verified in accordance with their medicinal uses, as the optimal maturity stage could be different depending on the target therapeutic function. In addition, it is also noticeable that mangiferin alone was less potent against bacteria than whole-leaf extracts, suggesting the important roles of other ingredients that co-exist in the raw materials. Further studies on this topic are therefore needed, with the purpose of identifying these compounds as well as their changes during the maturation process (and further elucidating the mechanisms involved). 

Vietnam is the 13th largest producer of mangoes in the world, with a total production area of 87,000 hectares [67]. During the cultivation of this tree, leaves are common by-products produced mainly through regular pruning activities [68]. In high-density planting of mangoes, this strategy is essential to ensure a well-balanced canopy with highly productive terminal shoots [68]. With several cycles of pruning occurring each year, enormous amounts of waste are generated from this activity, and leaves at various maturity stages have been identified as the main by-products [68,69]. These leaves could be applied as tea, flavorings, vegetables, food supplements, animal fodder, and herbs [68,69]; however, most of them are discarded as waste. However, because mango leaves have been observed to have highly therapeutic effects against bacteria, further studies should be carried out to exploit their potential in medicine as they could be beneficial for both pharmaceuticals and agriculture. In addition, our study suggests that the classification of leaves into different maturity levels is necessary to maximize their effects. Even though leaves of young ages only make up a small proportion and those of a dark reddish brown and yellow color are difficult to collect because they usually appear in only the first three to four weeks of the leaf-flushing period [68], our study showed that they were remarkably more potent in terms of antibacterial properties and therefore should be separately harvested for medical uses. However, further research to standardize in vivo applications is also necessary to optimize their therapeutic effects.

## 3. Materials and Methods

### 3.1. Plant Material and Extraction

**Figure 5:**
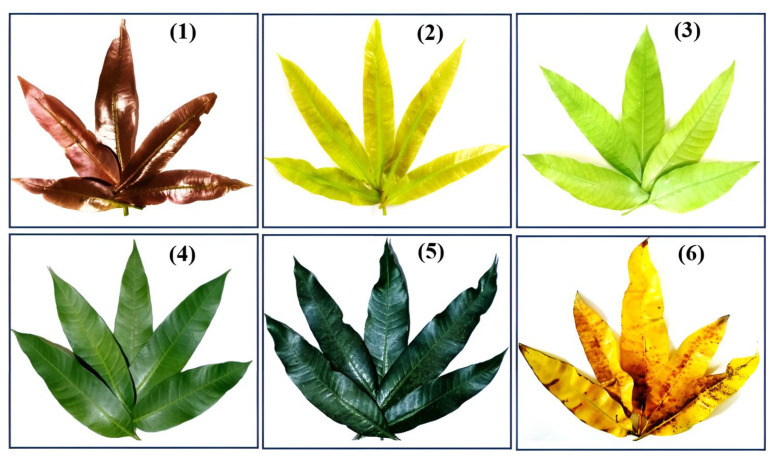
Photographs of typical mango leaves at various stages of maturation. (**1**): Young dark reddish brown leaves; (**2**): young yellow leaves; (**3**): young light green leaves; (**4**): mature green leaves; (**5**): old dark green leaves and (**6**): old yellow leaves.

Mango leaves were collected in Vuon Duoc Lieu Herbarium, Vietnam National University of Agriculture (Hanoi, Vietnam). The plant identities were confirmed by Dr Tho Thi Bui based on the voucher specimens that have been deposited at Vietnam National University of Agriculture. The classifications of mango leaves into different maturity levels were performed based on their colors, as described by Itoh et al. [12,14], Anbalagan et al. [34], and Ramírez et al. [70], with some modifications. Leaves were separated into the following groups: (1) young dark reddish brown leaves, (2) young yellow leaves, (3) young light green leaves, (4) mature green leaves, (5) old dark green leaves, and (6) old yellow leaves, as shown in Figure 5.

All leaf materials in the six groups were collected from the same plants during the spring season in Vietnam (March 2023). Because mangoes are evergreen trees, leaves at all maturity stages, characterized by the different colors, can be simultaneously generated during their leaf-flushing periods [14], which usually coincide with the spring or the wet season [68,69,70,71]. The fresh leaves were then washed and oven-dried at 50 °C in 96 h to obtain a constant weight. Dried materials were then ground into powder with a coffee blender before passing through a sieve with a nominal mesh size of 1 mm. Extractions were performed following our previous study [72] and with some modifications. Ethanol was selected as the solvent because it has been identified as the most preferable solvent for both antibacterial effects [50] and mangiferin extraction [34] from mango leaves. In brief, 10 g of powder was stirred with 300 mL of ethanol and left at room temperature for 24 h for absorbance. The mixtures were then filtered through 2 layers of cheese cloth, centrifuged at 10,000× *g* for 30 min, and finally passed through grade No. 2 qualitative filter paper (Advantec MFS Inc., Dublin, CA, USA) to remove all precipitates. Filtrates were then concentrated at 37 °C using a rotary evaporator at low atmospheric pressure to remove all solvents and obtained dried extracts. These final weights were then used to calculate extraction yields (%). All extracts were kept in a refrigerator at 4 °C for experimental analyses.

### 3.2. Reagents and Bacterial Strains

Mangiferin references at analytical standards (purity of ≥98%) were purchased from Sigma-Aldrich (St. Louis, MI, USA). HPLC-grade ethanol, methanol, and phosphoric acid at analytical levels were purchased from Merck (Darmstadt, Germany). Tested bacteria, including three Gram-positive (*Bacillus cereus* (*B. cereus*) ATCC 11778, *Bacillus subtilis* (*B. subtilis*) ATCC 6633, and *Staphylococcus aureus* (*S. aureus*) ATCC 25923) and three Gram-negative (*Escherichia coli* (*E. coli*) ATCC 25922, *Pseudomonas aeruginosa* (*P. aeruginosa*) ATCC 9027, and *Salmonella enterica* subsp. *enterica* serovar Typhimurium. (*S. typhimurium*) ATCC 13311), were purchased from the American Type Culture Collection (ATCC, Rockville, MD, USA). 

### 3.3. HPLC Analysis of Mangiferin

Analysis of mangiferin was performed using HPLC techniques, followed the “General instructions for the determination of flavonoid content by HPLC method”, which was established by the National Institute for Food Control and accredited by Vietnam Standards and Quality Institute (Code NIFC.05.M.235. Documentary number: 894.2020/QĐ-VPCNCL, issued 2020) [73], with some modifications. Briefly, the system consisted of an Agilent C18 (250 mm × 4.6 mm × 5 μm) column, which was connected to a 1260 Agilent HPLC (Agilent Technologies, Palo Alto, CA, USA) and equipped with a UV detector. The mobile phases were A: 0.1% acetic phosphoric in double-deionized water and B: methanol. The gradient conditions were as follows: solvent B: 0 min, 10%; 1 min, 10%; 5 min, 50%; 13 min, 70%; 16–20 min, 10%. Other chromatographic conditions were as follows: flow rate: 1 mL/min, column temperature: 30 °C and run time: 20 min. The wavelength of detection was 360 nm. The contents of mangiferin in samples were calculated by comparing the sample peak areas (% fluorescence) with those in the standard curve for mangiferin. HPLC analysis of extracts was performed in triplicate. 

### 3.4. Evaluation of Antibacterial Effects of the Extracts

The effects of extracts on bacteria were evaluated through broth dilution and agar well diffusion methods, following our previous study [74] and with some modifications. In order to observe dose-dependent effects, 10% dimethyl sulfoxide (DMSO) was applied to dilute extracts and mangiferin to obtain serially tested concentrations. 

The broth dilution method was performed to determine MIC values following the methods of the Clinical and Laboratory Standards Institute [75] and with some modifications to adjust to the necessary conditions for testing plant materials [74]. Tested solutions were mixed with Muller Hinton broth in 96-well microplate to produce serial dilutions ranging from 1000 μg/mL to 0.98 μg/mL. The final bacterial concentration was adjusted to 5 × 10^5^ cfu/mL. All bacteria were incubated at 37 °C for 24 h. The lowest concentration displaying no visible growth was recorded as the MIC. In addition, 10% DMSO served as a negative control and kanamycin was applied as a positive and quality control. Its MIC against *E. coli* ATCC 25922 was determined to be 2 μg/mL, which was within the acceptable limits (from 1–4 μg/mL) established by the Clinical and Laboratory Standards Institute [75].

The agar well diffusion method was performed following Nguyen et al. [74]. Briefly, a Muller Hinton agar plate was inoculated with bacteria at a final concentration of 10^6^ cfu/mL, and a hole with a diameter of 10 mm was punched aseptically with a cork borer. Then, 100 μL of tested materials, including extracts and purified mangiferin at established concentrations, were added into the well. Agar plates were incubated under 37 °C for 24 h and inhibitory zones (excluding 10 mm of well diameter) were measured. In these experiments, extracts were examined with concentrations starting at 2 mg/mL, while the concentration of mangiferin was 0.5 mg/mL. The 10% DMSO induced no inhibition and was applied as a negative control. Experiments were performed in triplicate. 

### 3.5. Statistical Analysis

Results are expressed as means ± standard deviation (SD). Statistical analyses were performed via an unpaired *t* test or the Tukey test after a one-way analysis of variance (one-way ANOVA). Significance was established when the probability level was equal to or less than 5%.

## 4. Conclusions

Our study, for the first time, has established the relationship between the mangiferin contents and antibacterial properties of mango leaves at six different stages of maturity. The results showed that leaves at young stages, characterized by color ranging from dark reddish brown to light green, were able to exert better activities against bacteria. The decrement in the antibacterial properties of mango leaves following the maturation process could be explained, at least in part, by the reduction in mangiferin contents. Our study highlights that mango leaves at young stages are better materials for treating bacterial infections. However, further studies are still necessary to confirm their therapeutic properties under in vivo conditions.

## Figures and Tables

**Table 1:** Contents of mangiferin (mg/g extract and mg/g dried powder) in mango leaves at different maturity stages.

Material	Extraction Yield (%)	Mangiferin Content (mg/g Extract)	Mangiferin Content (mg/g Dried Powder)
Young dark reddish brown leaf	26.39 ± 0.13 ^a^	310.06 ± 9.88 ^a^	81.82 ± 2.61 ^a^
Young yellow leaf	33.13 ± 1.04 ^b^	220.71 ± 3.72 ^b^	73.12 ± 1.23 ^b^
Young light green leaf	43.66 ± 0.40 ^c^	137.96 ± 2.45 ^c^	60.23 ± 1.06 ^c^
Mature green leaf	41.33 ± 0.07 ^c^	63.39 ± 2.55 ^d^	26.20 ± 1.05 ^d^
Old dark green leaf	42.48 ± 0.42 ^c^	31.46 ± 0.32 ^e^	13.36 ± 0.13 ^e^
Old yellow leaf	42.58 ± 0.71 ^c^	20.46 ± 0.35 ^f^	8.71 ± 0.15 ^f^

Results are expressed as means ± standard deviation (SD) of three tests. Values with different superscript letters (a to f) indicate significant difference (*p* < 0.05) compared with other values of the same column, assessed by one-way ANOVA followed by Tukey’s post hoc test.

**Table 2:** Minimum inhibitory concentration (μg/mL) of ethanol extracts of mango leaves at six maturity stages and mangiferin against bacteria.

Tested Material	Gram (+)			Gram (−)		
	*Bacillus cereus*	* Bacillus subtilis *	*Staphylococcus aureus*	*Escherichia coli*	*Salmonella* typhimurium	*Pseudomonas aeruginosa*
Young dark reddish brown leaf	7.81	15.63	1.95	7.81	31.3	125
Young yellow leaf	15.63	31.3	1.95	7.81	62.5	125
Young light green leaf	31.3	31.3	3.91	31.3	125	500
Mature green leaf	31.3	62.5	7.81	31.3	125	500
Old dark green leaf	62.5	125	7.81	62.5	250	1000
Old yellow leaf	250	500	125	250	500	1000
Mangiferin	7.81	15.63	3.91	7.81	31.3	62.5

**Table 3:** Inhibitory zones (mm) induced by ethanol extracts of mango leaves at different maturity stages and mangiferin on bacteria.

Bacterium		Material	Concentration (mg/mL)			
Gram-positive	*Bacillus cereus* ATCC 11778	Leaf extract	2 mg/mL	1 mg/mL	0.5 mg/mL	0.25 mg/mL
		Young red	15.6 ± 0.6 ^a,^*	10.8 ± 0.9 ^a^	4.7 ± 0.6 ^a^	-
		Young yellow	12.7 ± 0.5 ^a,^*	6.6 ± 0.8 ^b^	2.9 ± 0.2 ^b^	-
		Young light green	7.6 ± 0.6 ^c^	3.2 ± 1.4 ^c^	-	-
		Mangiferin	0.5 mg/mL	0.25 mg/mL	0.13 mg/mL	0.06 mg/mL
			7.7 ± 0.4	2.8 ± 0.6	-	-
	*Bacillus subtilis* ATCC 6633	Leaf extract	2 mg/mL	1 mg/mL	0.5 mg/mL	0.25 mg/mL
		Young red	13.9 ± 0.2 ^a,^*	10.5 ± 0.4 ^a^	-	-
		Young yellow	10.9 ± 0.3 ^b,^*	5.7 ± 0.5 ^b^	-	-
		Young light green	3.7 ± 0.6 ^c^	-	-	-
		Mangiferin	0.5 mg/mL	0.25 mg/mL	0.13 mg/mL	0.06 mg/mL
			5.7 ± 0.4	2.7 ± 0.5	-	-
	*Staphylococcus aureus* ATCC 25923	Leaf extract	2 mg/mL	1 mg/mL	0.5 mg/mL	0.25 mg/mL
		Young red	17.2 ± 0.9 ^a,^*	13.0 ± 0.2 ^a^	4.6 ± 1.2 ^a^	-
		Young yellow	13.6 ± 0.7 ^b,^*	9.6 ± 0.7 ^b^	2.8 ± 0.2 ^b^	-
		Young light green	10.1 ± 0.6 ^c^	6.9 ± 0.2 ^c^	2.3 ± 0.2 ^c^	-
		Mature green leaf	6.1 ± 0.4 ^d^	3.0 ± 0.2 ^d^	-	-
		Old dark green leaf	5.3 ± 0.4 ^e^	1.6 ± 0.5 ^e^	-	-
		Mangiferin	0.5 mg/mL	0.25 mg/mL	0.13 mg/mL	0.06 mg/mL
			9.3 ± 0.5	3.7 ± 0.6	-	-
Gram (−)	*Escherichia coli* ATCC 25922	Leaf extract	2 mg/mL	1 mg/mL	0.5 mg/mL	0.25 mg/mL
		Young red	14.8 ± 1.0 ^a,^*	9.9 ± 0.3 ^a^	6.2 ± 0.9 ^a^	-
		Young yellow	12.3 ± 0.8 ^b,^*	7.3 ± 1.0 ^b^	3.9 ± 0.5 ^b^	-
		Young light green	4.3 ± 0.7 ^c^	2.1 ± 1.0 ^c^	-	-
		Mangiferin	0.5 mg/mL	0.25 mg/mL	0.13 mg/mL	0.06 mg/mL
			6.8 ± 0.5	3.6 ± 0.5	-	-
	*Salmonella* typhimurium ATCC 13311	Leaf extract	2 mg/mL	1 mg/mL	0.5 mg/mL	0.25 mg/mL
		Young red	14.0 ± 0.5 ^a,^*	8.2 ± 0.2 ^a^	-	-
		Young yellow	11.2 ± 0.2 ^b,^*	6.0 ± 0.1 ^b^	-	-
		Young light green	3.5 ± 0.7 ^c^	-	-	-
		Mangiferin	0.5 mg/mL	0.25 mg/mL	0.13 mg/mL	0.06 mg/mL
			5.7 ± 0.4	3.0 ± 0.2	-	-
	*Pseudomonas aeruginosa* ATCC 9027	Leaf extract	2 mg/mL	1 mg/mL	0.5 mg/mL	0.25 mg/mL
		Young red	11.8 ± 0.5 ^a^	5.9 ± 0.4 ^a^	-	-
		Young yellow	9.6 ± 1.1 ^b^	4.6 ± 0.6 ^b^	-	-

Results are expressed as means ± standard deviation (SD) of three tests. Dashed lines indicate means no inhibition. Materials that showed no inhibitory zones at all tested concentrations are not shown. Values with different superscript letters indicate significant difference (*p* < 0.05) compared with other values from different extracts of similar concentrations on the same bacterium, assessed by a one-way ANOVA followed by Tukey’s post hoc test. Bold letters with asterisks indicate significant difference (*p* < 0.05) between the maximum inhibitory zones of whole extract vs. that of purified mangiferin, as assessed by an unpaired *t* test.

## Data Availability

All the data are shown in the manuscript and Supplementary Materials Data S1.

## Supplementary Materials

The following supporting information can be downloaded at: https://www.mdpi.com/article/10.3390/molecules29071443/s1. Supplementary Data S1: Mangiferin contents, extract yields, MICs and zones.

## Abbreviations

DMSO: dimethyl sulfoxide; Gram-positive: Gram (+); Gram-negative: Gram (−); MIC: minimum inhibitory concentration; RT: retention time; *B. cereus*: *Bacillus cereus*; *B. subtilis*: *Bacillus subtilis*; *E. coli*: *Escherichia coli*; *P. aeruginosa*: *Pseudomonas aeruginosa*; *S. aureus*: *Staphylococcus aureus*; *S. typhimurium*: *Salmonella enterica* subsp. *enterica* serovar typhimurium.
